# *In vitro* aneurysm models: a comprehensive review of 3D-printed, computational, and hydrogel platforms

**DOI:** 10.3389/fbioe.2026.1918602

**Published:** 2026-09-28

**Authors:** Saurav Ranjan Mohapatra, Christian Apel, Panagiotis Doukas, Alexander Gombert, Stefan Jockenhövel, Christian Uhl

**Affiliations:** 1 Department of Vascular Surgery, University Hospital RWTH Aachen, RWTH Aachen University, Aachen, Germany; 2 Department of Biohybrid and Medical Textiles Institute of Applied Medical Engineering, RWTH Aachen University, Aachen, Germany

**Keywords:** 3D printing, aneurysm, hydrogel phantoms, *in vitro* models, *in vitro* simulation, tissue engineering, vascular implant

## Abstract

The clinical translation of endovascular implants and new therapeutics for aortic aneurysms depend broadly on the reliability of the experimental testing models utilized. *In vivo* models present serious ethical and logistical limitations. Conversely, *in vitro* modelling is a high fidelity, human relevant approach, with carefully controlled environments and quicker clinical translation. Using the systematic literature search method, literature was screened for the various types of *in vitro* platforms used in aneurysm studies and classified into three main categories: (1) 3D-printed replicas, (2) computational fluid dynamics models, and (3) tissue mimicking hydrogel phantoms. A critical evaluation of material properties, fabrication techniques and experimental applications has shown that material compliance is crucial to reproduce physiological wall motion, and hydrogels provide outstanding properties for multi-modal imaging validation (Ultrasound, MRI and computed tomography) and for mimicking the structure of tissues. Moreover, the use of experimental flow visualization techniques also helps to overcome the challenges of disturbed hemodynamic patterns characterization. While several *in vitro* aneurysm models have achieved luminal endothelialization, the biomimetic incorporation of arterial smooth muscle cells together with endothelial cells in tissue-engineered aortic constructs is lacking in the literature and is an important future area of vascular research. To sum up, this review synthesizes these different *in vitro* modalities and their clinical translational potential, providing a clearer framework for future implant and therapeutic evaluation.

## 1 Introduction

Aortic aneurysms are permanent localized dilations of the aorta with a high risk for acute rupture and are associated with a high mortality rate (Leone et al., 2023). There are a number of clinically applied products such as the endovascular devices and targeted therapeutics, and ongoing product development is focused on improving clinical efficacy and reducing the historical limitations associated with the procedures (Troisi et al., 2023). As a result, new drugs and medical products are continually being created. A strong aneurysm testing platform is needed for assessing these new systems, however. *In vivo* animal studies are limited by ethical, financial and logistical challenges (Gertz et al., 2018/09), and *in vitro* models have proved to be a major impactful platform for controlled validation and preclinical testing of these biomedical innovations (Chen et al., 2021; Poupart et al., 2021; Patel et al., 2024; Jiao et al., 2026).

The *in vitro* aneurysm models should be able to mimic mechanical, biological and physiological conditions to provide clinically meaningful insights. A clinically appropriate aneurysm model must fit in three core pillars of fidelity: mechanical realism to capture non-linear wall elasticity and dynamic pressure buffering, biological compatibility to evaluate tissue interactions and cell responses, and mathematical/computational accuracy to solve complex, pulsatile fluid dynamics. Because no single experimental setup can capture all of these physiological parameters at once, researchers must choose platforms optimized for specific testing goals.

Modern *in vitro* studies have taken steps to more closely mimic the native anatomical variations, multi-directional biomechanical forces, and accurate tissue compliance present in an aneurysm. To address these demands, vascular modeling has evolved across three primary engineering paradigms: 3D-printed physical models, computational fluid dynamics (CFD) simulations, and tissue-mimicking hydrogel phantoms. Rather than operating in isolation, these modalities serve complementary roles spanning physical device evaluation, numerical prediction, and biomaterial synthesis.

### 1.1 3D printed in vitro models of aneurysms

Physical testing environments are essential to the evaluation of endovascular devices like flow-diverting stents, braided occluders and embolic coils that are required to deal with the complex geometry of human native arteries (Tsai and Savas, 2010). This is because the researchers can create physical vascular profiles using 3D printers, which can be directly observed by physical devices under controlled fluid conditions, to observe the deformity, deployment, and interaction with the vessel wall (Sodawalla et al., 2024). These 3D models represent a point of balance between the abstract computer models and the highly variable animal models, providing a repeatable platform to evaluate device performance, the placement of the structure and the trackability of the microcatheters prior to clinical use (Patel et al., 2024; Mamo et al., 2023; Celi et al., 2021).

Furthermore, multi-material 3D printing enables the simulation of different tissue properties within the aneurysm wall. 3D printed models are popular with the use in surgical planning, to rehearse procedures, including endovascular repair, and testing devices, such as measuring forces on stent-grafts. They also aid in the study of mechanical interactions such as forces exerted by embolization materials in the treatment of aneurysms (Lamano et al., 2025).

### 1.2 Numerical simulation and computational validation

Computational fluid dynamics (CFD) and fluid–structure interaction (FSI) are fundamentally numerical (*in silico*) methods but are included in this review because they are closely coupled with *in vitro* testing in modern benchtop systems, in which physiological boundary conditions for physical blood flow can be defined using computational approaches, and in turn, experimental flow data (e.g., PIV, 4D Flow MRI) are used to serve as ground truth for the validation of the numerical solvers.

While physical models allow for direct tactile and visual observation, computer programs provide high-resolution mapping of the invisible forces acting on the vascular wall (Saqr et al., 2020). CFD solves the fundamental equations of fluid motion to calculate velocity vectors, pressure gradients, and Wall Shear Stress (WSS) a primary mechanical driver of aneurysm growth and eventual rupture (Arzani et al., 2017; Salman et al., 2019).

Numerical programs allow researchers to test endless virtual modifications, such as changing an incoming flow angle or shifting a stent’s position, without the time and cost of physical manufacturing. For reliable production of data, virtual models need to be bounded by realistic physics (Chandra et al., 2013).

Systemic outflow networks: A time-varying velocity waveform is applied to the fluid going into the virtual model (Tsai and Savas, 2010). The outlets are connected to electronic-analog circuit models (Windkessel networks), which simulate the rest of the body’s circulation. The resistive parameters (R1, R2) and capacitive parameters (C) are combined in these circuits to represent the resistance and compliance of the arterial tree downstream of the pump (Thirugnanasambandam et al., 2021).

Non-Newtonian fluid realism: Blood behaves as a Newtonian fluid in high velocity streams, but is non-Newtonian, shear thinning in the stagnant areas of a wide aneurysm sac (Wang et al., 2024). If a computer program uses this simple Newtonian model here, it will underestimate the local fluid thickness and thus yield inaccurate calculations of the particle residence time and low-shear zones (Arzani et al., 2017; Wang et al., 2024).

### 1.3 Hydrogel frameworks and biomaterial modeling

Hydrogels represent an important class of biomaterials that mimic soft tissue compliance and acoustic properties of actual human blood vessels (Navindaran et al., 2023; Cloonan et al., 2014; Mix et al., 2018). The hydrogels are different from stiff silicones or plastics as they can absorb fluids and flex when under the physiological forces, similar to native vessel compliance (Hu et al., 2024; Thubrikar et al., 2001). This makes them invaluable for multi-modal imaging studies in which both structural deformations and fluid flow need to be monitored at the same time (Hariri et al., 2021).

Hydrogel-based models are used when it is important to replicate the mechanical and imaging properties of soft tissue. Polyvinyl alcohol (PVA) cryogels are commonly used because their stiffness and imaging characteristics can be adjusted through controlled freeze–thaw cycles. Such models are generally produced with moulds which are dissolved afterwards and result in realistic geometries of the vessels. Hydrogel phantoms are particularly useful because they can be used with a range of imaging methods, such as ultrasound, CT, and MRI (Little et al., 2022). They are particularly helpful for cross-checking with elastography using ultrasound, where the properties of certain materials are known, and the amount of tissue motion measured can be compared against their expected mechanical behavior (Mix et al., 2018).

*In vitro* simulation systems are aimed at simulating realistic blood flow. The setups are made with pulsatile flow loop and controlled pumps, imitating the physiological pressure and flow pattern. Blood like fluids are used for the viscosity matching to real blood. The use of advanced measurement techniques like particle image velocimetry allows detailed visualization of flow patterns by tracing fluorescent particles in transparent models (Özcan et al., 2023). These studies have revealed complex flow features such as vortices and stagnation zones, which are associated with thrombus formation and vessel wall weakening. They also demonstrate that wall shear stress induces aneurysm growth, and that the design and curvature of stent-grafts can impact forces that can cause complications such as endoleaks (Brand et al., 2023).

The purpose of this systematic review is to overview and classify the latest *in vitro* modeling of aneurysms in the three major areas: 3D printing, computational fluid dynamics, and tissue-mimicking hydrogels. It considers material compliance, fabrication methods and flow visualization approaches critically and highlights gaps in the literature for long-term biofidelic replication.

## 2 Materials and methods

To identify studies that focus on *in vitro* models of aneurysms, a systematic literature search was carried out. The search was broadly conducted from the inception of the databases in three main electronic databases (PubMed, Web of Science, and Scopus) for peer-reviewed articles published between 2000 and 2026. The literature search utilized a combination of Medical Subject Headings terms and targeted keywords. Key terms focused on *aneurysm* alongside *3D printing*, *in vitro models*, *hydrogels*, *computational modeling*, *biomimetic scaffolds*, and *hemodynamics*. Only peer reviewed articles in English language were included. The literature screening and selection followed the Preferred Reporting Items for Systematic Reviews and Meta-Analyses (PRISMA) 2020. Studies were eligible if they performed an *in vitro*/experimental biomechanical analysis of aneurysms, used 3D printed configurations, performed a computational fluid dynamics simulation that was validated by experiments using experimental loops, or involved the use of tissue-mimicking hydrogel phantoms and the analysis of physical hemodynamic parameters or aneurysm wall compliance.

On the other hand, the studies were excluded from the analysis if they involved only *in vivo* models, only numerical/computational simulation without validation *in vitro* or if they provided only isolated evaluation of healthy non-aneurysmal vascular segments or non-peer-reviewed literature types (abstracts, conference proceedings, patents, editorial reviews).

The quantification by literature is presented in detail in (Figure 1). An initial search of the literature was conducted and resulted in a total of 5,059 records (1,497 articles from PubMed, 1,759 from Web of Science and 1,803 from Scopus). A programmatic deduplication filter was applied before the screening phase, removing 3,802 duplicate records. To make sure there was a thorough and systematic selection process, three separate reviewers screened all articles retrieved according to predetermined inclusion and exclusion criteria, key research topics, target keywords and expected study results. After the initial screening, the final decision of the included studies was made by the senior authors, who also gave feedback on the screening process. The data from all studies included were systematically collected and captured in a standardized data template to describe the model type, material properties, flow parameters, key applications, and biological relevance. Of these, 1,257 unique records, 937 of these were excluded due to not meeting basic inclusion criteria. This resulted in 320 reports for full-text retrieval and 39 of these reports were not successfully retrieved due to institutional access restrictions or lack of published full text. We analyzed a set of 281 full-text reports and determined that they met the final eligibility criteria. During this secondary screening phase, a total of 233 reports were excluded because of detailed criteria: 165 had no relevant experimental outcomes; 52 were *in vivo* animal studies only; and 16 had inadequate data quality or incomplete reporting of methodology. After this screening cascade, 48 original studies met all the criteria and were selected for a qualitative data synthesis and included in the systematic review.

**FIGURE 1:**
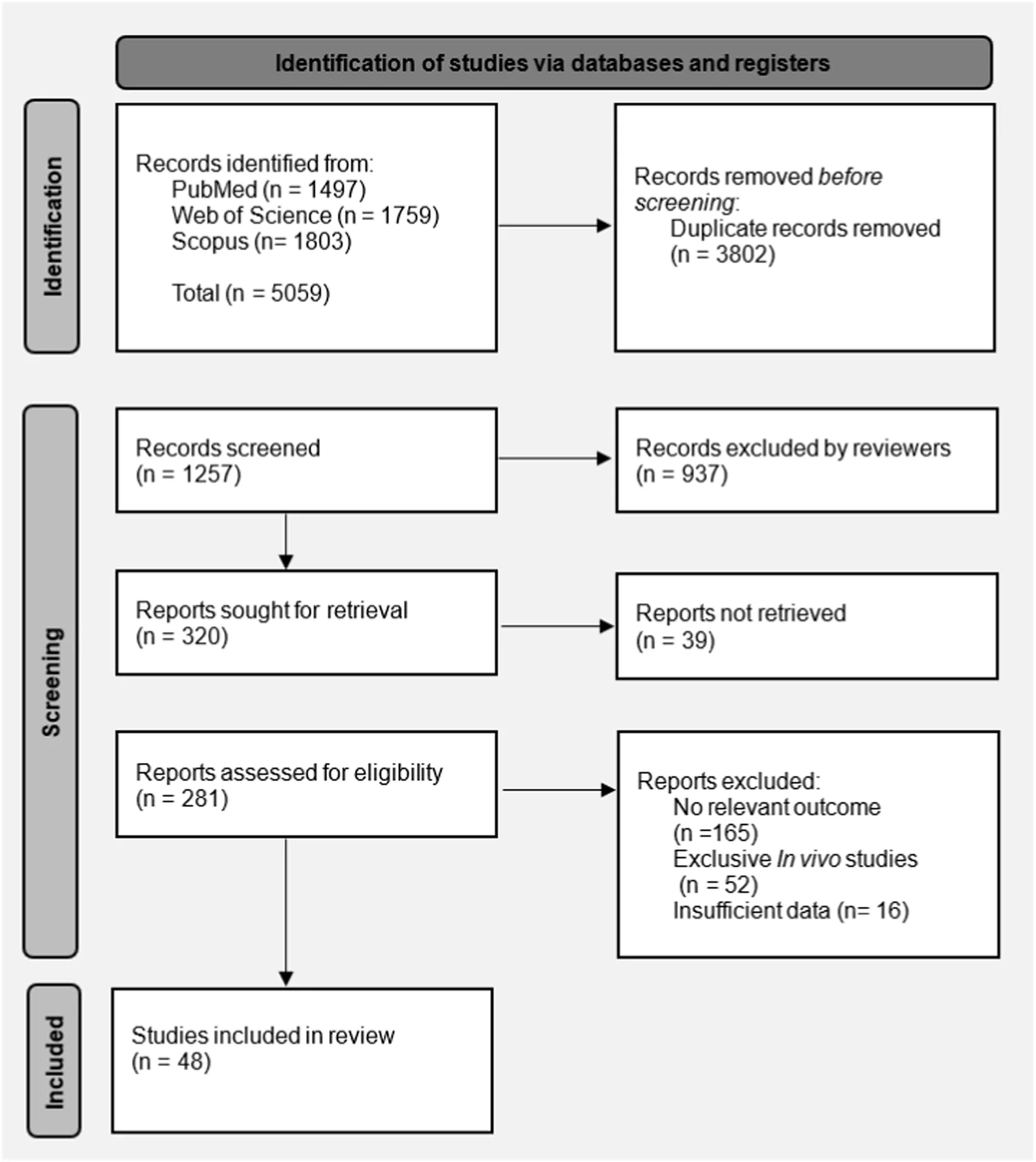
The systematic literature search, screening and selection process for *in vitro* aneurysm modelling studies are depicted on the PRISMA 2020 flow diagram.

To evaluate the 48 studies, we created five major functional domains. The domains included geometric and anatomical fidelity, biomechanical compliance matching, hemodynamic realism, experimental diagnostic validation and methodological reporting. Each domain was rated on a three-point scale of compliance (Low, Moderate, High) with the evaluations being synthesized during data extraction.

### 2.1 Quality assessment and methodological risk of bias

A methodological quality assessment (QA) was carried out to assess the internal validity of literature included. However, these traditional clinical risk of bias assessment tools were not suitable for the 48 selected studies as they only included *in vitro* engineering modalities and physical benchtop simulators, but not clinical human or animal trials. Instead, the methodological quality and internal validity of the studies included in the analysis was critically evaluated with custom designed engineering and experimental validation criteria.

## 3 Results

### 3.1 3D printed *in vitro* models of aneurysms

The transition of *in vitro* aneurysm modeling from glass tubes to 3D-printed, patient-specific models has enabled high-fidelity studies of hemodynamics, device efficacy, and rupture prediction (Ruiz de Galarreta et al., 2017). The reviewed studies were categorized by fabrication technique, materials, and clinical application.

Investigators use different approaches to fabrication of vascular networks:

Lost-core casting: This is a technique in which a water-soluble (often Polyvinyl Alcohol or PVA) sacrificial core is printed, which replicates the inner lumen of the blood vessel (Kumar and Sheet, 2026). The core is placed inside a casting mold, covered with an optically transparent elastomer such as Polydimethylsiloxane (PDMS) and cured (Antonuccio et al., 2023; Yazdi et al., 2019). After being submerged in the circulating water bath, the internal core is dissolved and a smooth, highly transparent hollow channel results which is ideal for optical flow diagnostics (Mirgolbabaee et al., 2025b).
Multi-material PolyJet printing: To mimic the non-uniform, elastic properties of real vascular tissue, multiple mass jetting can be used in the same build to deposit two or more different photopolymers. Through careful formulation of the rubber-like element to the stiffer polymer, the developer is able to print models with local deformation properties and directional variations to represent the diseased status of an aneurysm wall (Ruiz de Galarreta et al., 2017; Ruiz de Galarreta et al., 2016).
Vat photopolymerization: Direct Printing using flexible resins (e.g., Formlabs Elastic 80A) is suitable for the rapid production of complex vascular geometry with a very good dimensional accuracy (Yan et al., 2024; de Nys, 2025). The main trade-off is optical, as the refractive index of such cured resins may cause aberration with regular laser imaging, necessitating special inner coating or complex fluid matching to eliminate aberrations (Morita et al., 2023).

In summary, 3D printing methods deliver accurate patient-specific geometries for device testing, although matching native vessel compliance remains a key trade-off among printed resin formulations.

### 3.2 Numerical simulation and computational validation

*In vitro* simulation and hemodynamic analysis are fundamental to understanding aneurysm progression, validating numerical models, and predicting rupture risk (Table 1). Modern research leverages an integrated approach, combining Mock Circulatory Loops (MCL), Particle Image Velocimetry (PIV), and fluid–structure interaction (FSI) to replicate the complex pulsatile environment of the human vasculature (Salman et al., 2019).

**TABLE 1:** Numerical and experimental flow-loop simulations were classified by indicating core research categories, investigation modalities and target hemodynamic metrics.

Research category	Modality/technique	Key hemodynamic parameters	Author, year
Abdominal aortic aneurysm (AAA)	Integrated PIV and FSI	Velocity magnitude, wall displacement, Pearson correlation (PCC)	Özcan et al. (2023)
Intracranial aneurysm (IA)	High-speed video microscopy	Flow velocity, recirculation, vortex identification	Souza et al. (2023); Saqr et al. (2020)
Aortic dissection (AD)	Non-Newtonian CFD and test bench	Wall pressure (WP), WSS, reflux velocity	Wang et al. (2024); Birjiniuk et al. (2017)
Compliance modeling	FSI vs. CSS (1-way uncoupled)	Peak wall stress, energy dissipation	Salman et al. (2019); Chandra et al. (2013); Thubrikar et al. (2001)
Thrombosis modeling	Thrombin-fibrinogen flow system	Occlusion percentage, clot propagation area	Ngoepe et al. (2021); Stamatopoulos et al. (2010)
Vortex dynamics	Stereoscopic PIV/HLPT	Swirling strength, enstrophy, transverse velocity	Deplano et al. (2016); O’Leary et al. (2015); Finol et al. (2003)

#### 3.2.1 Fluid-structure interaction (FSI) vs. rigid models

Early computational models would employ fixed walls to reduce computation time but this would not allow for natural pulsation expansion of an artery and lead to inaccurate peak stress values (Alimohammadi et al., 2015). Fully coupled FSI simulations overcome this by computing the feedback loop continuously: the deformed wall geometry represents the elastic wall model, and the new geometry of the wall instantly changes the fluid path (Chandra et al., 2013; Özcan et al., 2023).

These dual-domain simulations are validated by comparing the Reynolds and Womersley numbers of physical tests with these numbers of the virtual tests, and by running physical fluid loops under identical conditions, Particle Image Velocimetry (PIV) is used to confirm that the virtual flow patterns are the same as the physical ones (Deplano et al., 2016; Etminan et al., 2022). Combining fully coupled FSI and non-Newtonian flow dynamics efficiently resolves local WSS and recirculation showcases that rigid-walled numerical models underestimate.

### 3.3 Hydrogel tissue-mimicking phantoms

Hydrogel tissue-mimicking phantoms represent a specialized class of vascular models designed to bridge the gap between rigid synthetic models and complex animal studies. These phantoms are engineered to replicate not only the anatomical geometry of aneurysms but also the mechanical compliance, acoustic properties, and biological environment of human tissue (Jang et al., 2020).

The main types of structural hydrogels can be processed into different shapes and forms by using a variety of different methods (Table 2). The most popular structural formulation is Polyvinyl Alcohol Cryogel (PVA-c) (Kumar and Sheet, 2026; Mackle et al., 2020). This is a hydrogel prepared without the use of harsh chemical cross linkers by dissolving the raw PVA powder in water. Rather, it is subjected to multiple freeze-thaw thermal cycles (Mix et al., 2018).

**TABLE 2:** Characterization of biomaterials in in vitro systems, including macromolecular composition, unique functional characteristics and multi-modal imaging compatibility.

Hydrogel system	Primary composition	Key research application	Unique functional feature	Author, year
PVA-c (Cryogel)	Polyvinyl alcohol powder (5–25% by mass)	Ultrasound Elastography validation	Tunable stiffness via freeze-thaw cycles and concentration.	Mix et al. (2018)
PEGDMA	Poly(ethylene glycol) dimethacrylate	Long-term embolic agent fatigue testing	Photopolymerizable *in situ*; withstood 5.5 million pulsatile cycles.	Poupart et al. (2021)
STB (Shear-Thinning)	Gelatin + Silicate Nanoplatelets	Endovascular occlusion (AAAs, VAAs)	Self-sustaining structure; injectable via catheter; non-adhesive.	Schroeder et al. (2022)
STB-DOX	STB + Doxycycline (0.3% w/v)	Bioactive embolization	Induced endothelial ablation and inhibited MMP activity.	Zehtabi et al. (2023)
Gelatin-Fibrin	Gelatin + Fibrinogen + Thrombin	Living “Blood Vessel Mimics” (BVMs)	Supports confluent endothelial lining; mimics brain tissue stiffness.	Jang et al. (2020)
Thrombin-Fibrinogen	Purified human plasma proteins	Clot growth tracking and visualization	Recreates biochemical coagulation cascade without whole blood.	Ngoepe et al. (2021)
Gel-wax	Mineral-oil based wax	Multimodality imaging (US/CT)	Wall-less phantom fabrication when used with water-soluble PVA cores.	Little et al. (2022); Maneas et al. (2018a)

The polymer microcrystals are formed each freezing cycle, which results in an increased stiffness of the material. The number of cycles and the use of contrast agents such as calcium carbonate can be used to finely tune the elasticity and ultrasound scattering properties of the gel, to match those of surrounding tissue structures (Kumar and Sheet, 2026).

#### 3.3.1 Functional and injectable embolic hydrogels

Embolic Hydrogels are Functional and Injectable Hydrogels. In addition to the ability of mimicking host tissue, engineered specialized hydrogels have been utilized as injectable therapeutic agents to deliberately obstruct the flow within the aneurysm sac (Poupart et al., 2021; Rodriguez et al., 2015).

Shear-thinning nanocomposites: The formulations are based on a reversible network of polymer chains (such as gelatin) combined with synthetic silicate nanoplatelets (Zehtabi et al., 2023). The internal bonds temporarily dissolve in the presence of the high shear stress of pushing the gel through a thin surgical catheter, so that the gel can flow like a liquid. As soon as it is released into the low-shear environment within the aneurysm sac, the bonds snap back together, and the material becomes solid and stable, thus blocking blood flow into the sac (Schroeder et al., 2022; Zehtabi et al., 2023).

Photopolymerizable formulations: The materials inserted into the aneurysm are supplied in a liquid form, with the precursor of the material being a polyethylene glycol dimethacrylate (PEGDMA) compound that is combined with contrast dyes (Poupart et al., 2021). A microcatheter with an internal optical fibre is introduced into the area and delivers blue-light illumination (e.g., 405 nm) to the target. This activates a photoinitiator that quickly cross-links the liquid into a durable, conforming elastic matrix which resists continuous pulsating flow without breaking apart. The most sophisticated *in vitro* models are made into biohybrid Blood Vessel Mimics (BVMs) by seeding living human endothelial cells inside the fluid channels (Jang et al., 2020; de Nys, 2025). In continuous flow conditions, these cells will be aligned with the fluid flow, resulting in a living barrier that can mimic the condition of a real vascular healing (Hynes et al., 2020; Ngoepe et al., 2021). Overall, hydrogel models combine soft-tissue compliance, imaging precision, and cellular seeding to accomplish high functional biofidelity amongst benchtop models.

## 4 Discussion

The articles screened and analysed in this review shows that the study of *in vitro* aneurysms is quickly progressing towards full biofidelic simulation. Advances in biomaterial synthesis, additive manufacturing, and high fidelity fluid-structure interaction (FSI) are increasingly being confronted together to capture native vascular compliance and physiological hemodynamics. The *in vitro* platforms are reviewed in terms of the three main paradigms: 3D-printed replica models, computational/*in silico* models, and tissue-mimicking hydrogel phantoms, with the aim of offering a structured review. The three technologies are not standalone paradigms, but rather complementary elements of a cohesive multiscale modeling approach. The patient-specific imaging data (DICOM) is initially processed in computational solvers to map hemodynamic stressors, such as low time-averaged wall shear stress and elevated endothelial cell activation potential, which are associated with high risk. These computational insights have been directly translated to material selection and physical architecture of multi-material 3D-printed phantoms and bioprinted hydrogel matrices. Lastly, the bio-functionalized living constructs are dynamically flow conditioned in mock circulatory loops to afford a robust experimental validation using particle image velocimetry (PIV) or echoPIV for iteration of initial computational boundary conditions. To conceptualize these modalities as an iterative and integrated pipeline helps to put a mechanical testing of benchtop materials into the context of a complex biological pathophysiology. The key materials, advantages, critical engineering bottlenecks and translational potential of these three modeling approaches are summarized in (Table 3).

**TABLE 3:** Overview and critical comparison of in vitro aneurysm modeling paradigms.

Modeling paradigm	Primary substrates and technologies	Key advantages	Critical engineering bottlenecks	Translational and clinical potential
3D-printed replicas	SLA/FDM photopolymers, silicone (PDMS), lost-core casting	• High patient-specific geometric fidelity from DICOM data • Excellent optical transparency for PIV flow mapping • Reusable and durable for benchtop loops	• High wall stiffness relative to native tissue • Isotropic mechanical response • Lacks cellular dynamics and wall remodeling	• High for interventional surgical training • Device deployment testing • Benchtop PIV flow validation
Computational models	Finite element analysis (FEA), CFD, 1-way CSS, 2-way FSI coupling	• High-resolution mapping of WSS, OSI, and ECAP • Non-invasive prediction of high-risk rupture sites • Rapid virtual interventional planning	• High computational cost for 2-way FSI • Linear elasticity assumptions overestimate deformation by >27% • Synthetic loop impedance mismatches	• High for patient-specific risk stratification • Hemodynamic forecasting • Virtual stenting/coiling evaluation
Hydrogel phantoms	PVA-c, GelMA, fibrin, PEGDMA nanocomposites, cell-laden matrix	• Tunable tissue-like compliance and hyperelasticity • Acoustic and radiological artifact-free imaging • Supports luminal EC/SMC co-culture	• Poor long-term shelf life and structural degradation • High swelling kinetics (up to 151%) distort geometry • Complex fabrication and handling	• Highest for disease mechanism modeling • Cellular mechanotransduction • Targeted drug delivery testing

### 4.1 3D-printed replicas

A critical review of 3D models shows that there is an inherent trade-off between geometric accuracy, material compliance and optical transparency when creating replicas for bioengineering. While SLA and FDM techniques accurately reproduce patient-specific geometries, direct-printed photopolymers remain significantly stiffer than native tissue (Özcan et al., 2023). On the other hand, optical opacity is solved by lost-core sacrificial casting techniques (PVA-core) but not all of the above-mentioned techniques are able to capture the non-linear and hyperplastic wall dynamics. The findings from (Table 4) show that additive manufacturing has revolutionized the field of surgical planning and endovascular device tracking. One of the best features of today’s 3D printing technology is the ability to re-create a complex and tortuous geometry directly from clinical DICOM data (CT and MRI scans). But a careful examination of the literature shows that there is a fundamental operational compromise between geometric accuracy, flexibility of the materials, and optical access. This bottleneck has been addressed by several investigations using very innovative casting methods. Vega et al. (2025) and Antonuccio et al. (2023) printed very interesting work using water soluble Polyvinyl Alcohol (PVA) for printing sacrificial internal lumen cores that were later encapsulated with Polydimethylsiloxane (PDMS). This lost-core technique provides an optically transparent, fluid-tight hollow channel suitable for Particle Image Velocimetry.

**TABLE 4:** Overview of printing methods, material choices, and key applications.

Fabrication technique	Primary materials	Key application	Author, year
Lost-core casting	PVA (core)/PDMS (vessel)	PIV; FSI validation; device deployment	Vega et al. (2025); Antonuccio et al. (2023); Özcan et al. (2023)
Lost-mold casting	PVA or ABS (mold)/PDMS (vessel)	Surgical planning; hemodynamics	Antonuccio et al. (2023); Celi et al. (2021)
Direct PolyJet printing	Agilus30; FLX Digital Materials	Anisotropic tissue mimicry; rupture modeling	Ruiz de Galarreta et al., (2017); Ruiz de Galarreta et al. (2016); Sodawalla et al. (2024)
Direct resin printing (SLA)	Flexible 80A; Elastic V2 Resins	echoPIV; microCT; multimodality imaging	Elmer et al. (2025); Mirgolbabaee et al. (2025a)
3D bioprinting	Gelatin-Fibrin Hydrogel; hCMECs	Living “Blood Vessel Mimics” (BVMs)	Jang et al. (2020); Hynes et al. (2020)
Wall-less/hybrid Modeling	PVA (core)/TMM (soft tissue mimic)	Multimodality imaging (CT/US/MRI)	Little et al. (2022)
Leached SMP	Shape Memory Polymer (SMP)	Endovascular Sac occlusion	Vega et al. (2025)

For each research goal, physical models are designed within three different geometric frameworks:

Idealized formulations: Use simplified, symmetrical geometries (straight tube with spherical side-wall bulges) (Stamatopoulos et al., 2010). They select specific parameters such as the width of the neck or the volume of the sac to derive parameters that describe the fluid properties at baseline without anatomical noise (Finol et al., 2003).
Patient-specific architectures: Extracted directly from clinical CT or MR angiography, these models retain the authentic irregularities of a specific patient’s anatomy, including extreme tortuosity and localized wall thinning (Patel et al., 2024; Özcan et al., 2023).
Cohort based averaged models: These are models generated by mathematical averaging of vascular data from a large number of patients, eliminating individual anomalies and thereby providing a model for product verification which is statistically representative of the population (Mirgolbabaee et al., 2025b).

#### 4.1.1 Fabrication accuracy and methodology

There is a definite trade-off between surface finish and casting cost between the use of lost-mold and lost-core casting. The average dimensional deviation of lost-mold casting is −0.2 mm, and the surface inside the casting is smooth enough to reduce the optical noise in Particle Image Velocimetry (PIV) (Celi et al., 2021; Antonuccio et al., 2023). On the other hand, lost-core casting with consumer grade FDM printers and water-soluble PVA materials is very inexpensive and is capable of creating complex geometries that are difficult or even impossible to detach from a rigid mold. Lost-core casting was employed for the production of complex undercut geometries, such as those expected to produce the elastomeric vessel constructs, which would not be possible without damaging the cast construct by using traditional rigid molds (Özcan et al., 2023; Vega et al., 2025).

#### 4.1.2 Material property matching and bio-fidelity

One of the main problems in in vitro vascular modelling is the simulation of mechanical complexity in human tissues. Conventional PDMS (Sylgard 184) models can also be significantly stiffer than tissue, with a normalized compliance of ∼1.3% that of the human aorta, which may cause supraphysiologic pressure artifacts in benchtop flow loops (Schroeder et al., 2022). Next-generation technology for PolyJet (aka the “printed phantom” method) overcomes this limitation by printing composite digital structures in which elastic digital fibers are strategically integrated into a softer matrix to achieve a printed phantom that can accurately replicate the direction-dependent stiffness of human abdominal aortic aneurysms (Cloonan et al., 2014; Ruiz de Galarreta et al., 2016). In the case of hybrid fabrication, the high pouring temperatures necessary for casting and de-bubbling surrounding tissue-mimicking hydrogels are ideal for the more advanced SLA photopolymer resins, such as the Elastic V2, which have a tensile modulus of 12 ± 3.2 MPa, and are thermally stable up to 250 °C (Elmer et al., 2025).

#### 4.1.3 Functional, rupture, and biological modeling

An important step forward from the static silicone phantoms has been achieved in recent advances in additive manufacturing, which has allowed for the development of rupture models that can be predicted. Researchers can use these direct printing elastomers, known as Agilus30, (with a 30 Shore A hardness) to create giant aneurysm phantoms that reliably fail at about 850 cycles (12 min) under the neurovascular pressure and provide reliable baseline data for testing the ability of novel endovascular devices and shear-thinning biomaterials to stabilize the wall against imminent rupture (Sodawalla et al., 2024).

Building on this, 3D bioprinted aneurysm platforms are the most recent advancement as living cellular elements are added to the pipeline. These innovative models are distinguished by their unique use of a gelatin-fibrin hydrogel, which allows the growth of a confluent human cerebral microvascular endothelial cell lining, whereas mechanical models do not use such advanced constructs. In contrast to purely mechanical models, these advanced constructs use a customized gelatin-fibrin hydrogel for growth of a confluent human cerebral microvascular endothelial cell lining (Jang et al., 2020; Hynes et al., 2020). The living models enable real-time monitoring of plasma clotting and biochemical cascades, and the propagation of the clots was seen to occur in the vicinity of interventional devices (e.g., platinum embolization coils) but not due to the introduction of the synthetic phantom material itself (Jang et al., 2020).

#### 4.1.4 Imaging compatibility

The choice of the imaging modality is a major factor in determining material selection. The acoustic properties such as the speed of sound and attenuation of flexible photopolymer resins have been well characterized, making them ideal for flow field quantification via ultrasound (echoPIV) measurement (Mirgolbabaee et al., 2025a; Morita et al., 2023). Wall-less phantoms incorporated in gel-wax allows to completely avoid the radiological artifacts and artificial boundaries usually generated by the thickness of synthetic silicone walls, enabling the realistic image fusion during simulated surgical training for multimodality studies such as MRI and CT (Little et al., 2022; Maneas et al., 2018a).

### 4.2 Computational models

For numerical and benchtop hemodynamic models, selection should be based on a balance between computational load and physical realism. One of the important results from the hemodynamic studies is the large error resulting from assuming a rigid wall. In rigid models, CFD can give a basic outline of the flow patterns, but does not include the impact of the Windkessel effect, which involves the vessel wall acting as an energy reservoir (Salman et al., 2019; Finol et al., 2003). Transitioning from rigid models to uncoupled 1-way solid stress calculations (CSS) reduces computational times relative to full fluid-structure interaction (FSI), but underestimates true wall stress by up to 30% due to the lack of dynamic coupling between the deforming wall and the fluid jet (Scotti et al., 2005). During systole, the compliant wall distends and during diastole it recoils, generating elastic energy that is used to push vortices forward toward the distal end, which does not happen in rigid configurations (Scotti et al., 2005).

#### 4.2.1 Scaling, dynamic similarity, and advanced risk markers

Models must be dynamically similar, which requires matching dimensionless numbers in order to obtain clinically relevant results on the benchtop (Salman et al., 2019). Intracranial aneurysms have a Reynolds number between 100 and 750, while the peak Reynolds number for abdominal aortic aneurysms can be as high as 4,000 (Gobin et al., 2014). The Womersley number (α) indicates the pulsatility of the flow; when α < 4 the velocity profile is parabolic and when α > 10 the velocity profile is flat with rapid acceleration periods. To simulate physiological Wall Shear Stress (WSS) both parameters need to be matched (Crawford, 1959).

In addition to just velocity, researchers use derived parameters to indicate sites that are vulnerable to intraluminal thrombus (ILT) and rupture. These are measured as the Oscillatory Shear Index (OSI), a measure of the unidirectionality of shear stress, and the Endothelial Cell Activation Potential (ECAP), which is the ratio of OSI to Time-Averaged WSS (TAWSS). High-risk areas for wall inflammation and ILT growth can be identified by a critical ECAP threshold of 1.4 Pa −1 with a wide consensus (Arzani et al., 2017). In acute pathology (e.g., aortic dissection), the pressure in the false lumen can increase abruptly to +320 mmHg, creating large pressure gradients that induce larger and faster refluxes, and can further cause tissue tears (Wang et al., 2024; Birjiniuk et al., 2017).

#### 4.2.2 Integrated experimental validation and limitations

The combination of PIV and 4D MRI with numerical simulations offers a powerful tool for clinical planning validation (Thirugnanasambandam et al., 2021). The high fidelity PIV setup has shown a very good correlation with the FSI model for a Pearson coefficient of 0.85 (Özcan et al., 2023). This facilitates virtual interventional planning, with the ability to predict both device occlusion and biological clotting responses, which are very dependent on flow rates (e.g., occlusion is better with a flow rate of 40 mL/min than 80 mL/min because of the limitations on the residence time of the thrombin in the device) (Ngoepe et al., 2021).

These are all very high correlations, but there are still technical problems. The simplified assumption of linear elastic material properties is often used to describe wall deformation deviations greater than +27%, which is evidence of the hyperelastic, non-linear properties of aged or diseased aortic tissue (Yazdi et al., 2019; Meyer et al., 2011). Thus, while fully coupled 2-way FSI integrated with physical circulatory loops represents the gold standard for capturing dynamic wall recoil and localized hemodynamic markers (OSI, ECAP), its widespread benchtop adoption remains hindered by the technical challenge of impedance matching in synthetic loops, which often require specialized Windkessel modules to prevent supraphysiologic pressure artifacts (Thirugnanasambandam et al., 2021; Schroeder et al., 2022).

### 4.3 Hydrogel phantoms

One of the key benefits of hydrogel phantoms is their ability to be mechanically tunable. The contribution of Mix et al. (2018) is very relevant in the field of structural compliance and diagnostics. They showed the material’s elasticity and ultrasound scattering properties could be finely tuned without the use of harsh chemical cross-linkers, and showed they could be achieved by just repeating freeze-thaw thermal cycles that carefully control the material’s internal structure. This renders PVA-c phantoms the ideal benchmark for the validation of ultrasound elastography and comparison of physical tissue motion to predicted mechanical response. In addition to structural replication, some of the most remarkable and innovative advances in this dataset relate to the development of functional embolic hydrogels suitable for the validation of therapeutic devices that are meant to be injected into humans. Poupart et al. (2021) synthesized a photopolymerizable Poly(ethylene glycol) dimethacrylate (PEGDMA) framework. This hydrogel was introduced into the body using a microcatheter inserted through optical fibre and then cross-linked *in situ* using blue-light illumination, withstanding more than 5.5 million pulsatile cycles of embolic sac occlusion, a good sign of long-term durability. Likewise, Zehtabi et al. (2023) and Schroeder et al. (2022) developed ingenious nanocomposites (STB) which were shear-thinning and loaded with bioactive molecules such as Doxycycline. These biomaterials are liquefied when subjected to the high shear stress of a surgical catheter and promptly solidify when a catheter is withdrawn, and thus, *in vitro*, demonstrate endothelial ablation and matrix metalloproteinase (MMP) inhibition.

#### 4.3.1 Imaging compatibility and material constraints

This is because the choice of hydrogel is strongly dependent on the imaging technique in use. Reliable characterization of the speed of sound and attenuation properties of the resin are important for using it for ultrasound in echo PIV (Mix et al., 2018; Morita et al., 2023). and this makes PVA-c or flexible resins more preferred. Gel-wax is noted as a temperature-stable, water-insoluble tissue-mimicking material to be used for CT and MRI. Researchers have printed a 3D replica of a human aorta using a water-soluble PVA resin, encased it in a gel and then dissolved away the core to create a vessel with no walls that yields a high-fidelity computed tomography (CT) image and an ultrasound image without any synthetic boundary artifacts (Little et al., 2022; Maneas et al., 2018b).

Hydrogels are the best bio-fidelity but they also have their limitations when compared to silicone. They will usually expire sooner because they lose water or may get moldy. Moreover, some materials, such as PEGDMA 20 kDa, exhibit over-voluminizing responses up to 151%, which may result in material extrusion into the parent artery; other materials, like PEGDMA 6k-20% have very high swelling pressures and could potentially cause ruptures in the aneurysm wall during the curing process (Poupart et al., 2021; Alimohammadi et al., 2015).

Lastly, the idea of a true biological fidelity is best embodied by two groups who have developed living “Blood Vessel Mimics” (BVMs)—Jang et al. (2020) and Hynes et al. (2020). They even managed to bioprint gelatin-fibrin hydrogels and then seed it with human live cells, with which they were able to create a confluent endothelial lining that lines up under fluid flow. Together with the thrombin-fibrinogen coagulation loops they designed in 2021, this work represents another major advance in the modeling of *in vitro* vascular processes that fully replicate the complex process of vascular healing and the propagation of thrombus over time (without the use of animal models and systemic clotting complications).

#### 4.3.2 Translational bioengineering roadmap: cell integration, ECM remodeling, and living constructs

Physical phantoms (e.g., PDMS or 3D-printed photopolymers) offer excellent mechanical and hemodynamic fidelity but are lacking in cellular dynamics that would provide insight into the disease process and biological wall remodeling. Recently, the feasibility of functional smooth muscle cell (SMC) integration was elegantly demonstrated in ‘vessel-on-a-chip’ models with human induced pluripotent stem cell (hiPSC)-derived vascular SMCs or patient-derived vascular SMCs for the evaluation of pharmacological interventions (e.g., metformin therapies) and mitochondrial dynamic pathways in aortic aneurysm models (Ma et al., 2022; Abudupataer et al., 2021). One challenge yet to be overcome in existing research is scaling cellularized micro-architectures to macro-scale perfused 3D hydrogel phantoms (3D models) (Zhang et al., 2021).

For future vascular tissue engineering to move away from static mechanical models towards bio-inspired, living 3D constructs, four areas of bioengineering need to be emphasized. (1) *Multicellular co-culture frameworks:* Going beyond single layer endothelialisation; creating co-cultures where a confluent endothelial cell (EC) monolayer is seeded onto a bioactive hydrogel (e.g., GelMA, fibrin or hyaluronic acid blends) containing circumferential-aligned medial smooth muscle cells (SMCs) (Gao et al., 2022/08; Mohapatra et al., 2022). (2) *Patient-derived and hiPSC sourcing:* Use of hiPSC-derived vascular SMCs to represent patient-specific gene expression, inflammatory pathways, and variance in response to new drugs (Ma et al., 2022; Abudupataer et al., 2021). (3) *ECM remodeling and enzymatic degradation:* Using cell-degradable, matrix metalloproteinase (MMP)-sensitive hydrogel networks to mimic localized elastolysis, collagen degradation (MMP-2/MMP-9 turnover) and progressive weakening of the wall characteristic of expanding aneurysms (Polacheck et al., 2013). (4) *Bioreactor perfusion and mechanotransduction:* Using bi-axial cyclic radial stretch and dynamic fluid shear stress in bioreactor to maintain mature SMC contractile phenotype and to promote extracellular matrix synthesis under physiological flow conditions (Mohapatra et al., 2022; Rama et al., 2022/04). Solving these limitations will move *in vitro* aneurysm modeling from basic benchtop testing rigs into dynamic, living diagnostic tools that assess cell-mediated wall degeneration, and even preclinical regenerative therapies.

One drawback of the current literature is, however, the lack of combination of both endothelial cells and the native aortic wall microenvironment and tissue engineered models. While only luminal endothelialization has been demonstrated in hydrogel channels, incorporating functional arterial smooth muscle cells (SMCs) into the medial layer of the same model remains a major technical bottleneck. SMCs are also involved in the basic functions of remodeling extracellular matrix, regulating vascular tone, inflammatory signaling, and the progression of aneurysms. As a result, many existing tissue engineered aneurysms models are not physiologically relevant nor biomimetic, when SMC integration is not included. Creating multi-layered vascular constructs with endothelial cells and arterial SMCs may offer a more representative model to investigate the initiation, progression, wall degeneration of aneurysm and therapeutic response. In addition, such biomimetic models could increase the translative potential of preclinical vascular research and help to better assess novel endovascular interventions and regenerative therapies. It is therefore necessary to investigate further the integration of SMCs together with endothelial cells into engineered aortic scaffolds to establish a more natural vascular architecture and cell-cell interactions.

Although *in vitro* aneurysm models have been developed in recent years, there are several challenges in the bioengineering of these models that limit their clinical applications. The first limitation for scaling up microfluidic device models to 3D perfused aneurysm models at a macro-scale is the nutrient diffusion boundary, since nutrients cannot reach the center of the model within a large, elongated, or hollow structure. Secondly, during prolonged culture, hypoxic core necrosis may occur at a macro-scale, which is not observed in micro-scale models. Second, achieving long-term mechanical stability is still a challenge, due to high swelling kinetics of hydrogels and structural degradation in dynamic pulsatile flow. Maintaining wall integrity for conditioning in the multi-week bioreactor without geometric change or rupture is essential to ensure that a representative mechanotransduction is captured. Also there is batch-to-batch variability in natural polymers (such as fibrin and gelatin) and varying seeding densities of cells, which makes experiments less reproducible. To achieve reliable and repeatable devices for testing and drug screening, it will be crucial to standardise biofabrication procedures and to develop synthetic-natural hybrid hydrogels.

## 5 Conclusion

Advances in in vitro aneurysm modeling have changed from rigid tubes to highly compliant patient-specific platforms. This review illustrates that in order to understand the progression of aortic aneurysms, to develop our understanding of surgery, and to optimize surgical outcomes in the real world, the biomechanical environment of the human aorta must be captured. With direct multi-material 3D printing and sacrificial lost-core casting using PDMS we can produce tissue heterogeneities, non-uniform wall thicknesses and physiologic compliance features that mimic complex biological structures. Meanwhile, tissue-mimicking hydrogels in particular, PVA cryogels, have eliminated long-standing imaging barriers. Now, it is possible to compare hemodynamics across ultrasound, MRI and CT modalities, without losing image quality.

*In vitro* aneurysm models provides a very accurate platform for EVAR planning and practice of complex endovascular aortic repairs. Combined with fluid–structure interaction (FSI) simulations, these physical mock circulatory loops and 3D Particle Image Velocimetry (PIV) setups allow to map out the exact fluid behaviors, such as near-wall stagnation and exposure time to wall shear stress, which cause graft migration or endoleaks for the first time.

There are advantages and disadvantages to each modeling approach. Three-dimensional printed models are very accurate at anatomical detail but can be lacking in mechanical properties. Hydrogel phantoms have good tissue-like properties and imaging compatibility, but have a tendency to deteriorate over time and are difficult to fabricate. Technically complex and costly to set up, flow simulation models offer in-depth information about hemodynamics.

Although there have been advances towards the development of *in vitro* aneurysm models, some significant challenges still exist in creating a more complex tissue model of the native aortic wall. While the endothelialization was included in some studies, the incorporation of arterial smooth muscle cells together with endothelial cells(ECs) is still missing in most of the tissue engineered and biomimetic models. The combination of SMCs and ECs in future vascular constructs could significantly increase the physiological relevance of these platforms and aid in the understanding of the biology of aneurysms, disease progression, and therapeutic testing. Thus, the next step in the research of vascular tissue engineering is to develop fully biomimetic, multilayered aneurysm models.

Finally, the combination of realistic physical phantoms and validated computational models puts a new standard in vascular research. These patient-specific, predictive insights will put clinicians one step closer to therapies that are specific to each person’s biomechanical environment.
